# Effect of intradialytic resistance exercise on quality of life of hemodialysis patients in Hamad Medical Corporation: A quasi-experimental study

**DOI:** 10.5339/qmj.2026.33

**Published:** 2026-06-15

**Authors:** Jenine Joseph, Rawia Ali Amin Sa’abneh, Maryam Salem E. M. Al-Abdulla, Jude Xavier Anthony, Saji Philip, Sheena Mariam Oommen, Joseph Andog Macalimbon, Aileen Siapo Valdes, Manruel S. Punay, Alaedine Shurrab, Samia Ait Faqih, Kalpana Singh

**Affiliations:** Department of Nursing and Midwifery Education, AL Khor Hospital, Hamad Medical Corporation, Doha, Qatar; Department of Nursing and Midwifery- Dialysis Unit, AL Khor Hospital, Hamad Medical Corporation, Doha, Qatar; Department of Physiotherapy, AL Khor Hospital, Hamad Medical Corporation, Doha, Qatar; Department of Nephrology/Medicine, AL Khor Hospital, Hamad Medical Corporation, Doha, Qatar; Nursing & Midwifery Research Department, Hamad Medical corporation, Doha, Qatar

**Keywords:** Quality of life-4, NKF KDOQ, ESKD, WHO QoL -BREF, QoL

## Abstract

**Background:**

Dialysis patients suffer from anemia, muscle fatigue and atrophy, decreased aerobic capacity resulting in reduced physical activity, and decreased quality of life (QoL). The end-stage kidney disease patients often experience reduced QoL due to physical and psychological burdens. This study aims to evaluate the effect of intradialytic resistance exercise (IRE) on QoL, dialysis adequacy, muscle cramps, and physical function of dialysis patients.

**Methodology:**

A quasi-experimental study was conducted at Al Khor Dialysis Unit. Participants performed supervised TheraBand exercises during dialysis, including warm-ups, IREs, and stretching. WHO QoL – BREF (World Health Organization Quality of Life- (Brief Version)) questionnaires and physical function tests, including a 10-m walk test and a 30-second sit-stand test, were performed. The exercise was performed within the first 2 hours of the dialysis session for 6 months. Twenty-four patients were followed for a period of 6 months.

**Results:**

The mean QoL score at baseline was 67.9 ± 20.9, which increased to 70.3 ± 17.2 after 3 months and 74.5 ± 15.8 after 6 months. There were 71% male and 29% female in the average age group of 59.9 years. No significant correlations were observed between QoL and demographic factors such as gender, education, or income. Additionally, muscle cramps improved slightly from 4% at baseline to 8% post intervention. Kt/V, a measure of dialysis adequacy, remained stable after the intervention. Physical performance improved, with sit-to-stand test repetitions increasing from 7.2 ± 2.9 to 10.1 ± 4.5, though the time for the 10-m walk test improved slightly.

**Conclusion:**

The resistance exercise intervention demonstrated modest numerical improvements in quality of life and physical function, although these trends should be interpreted cautiously.

## 1. INTRODUCTION

End-stage kidney disease (ESKD) patients receiving hemodialysis experience substantial physical and psychological burdens that negatively affect their quality of life (QoL). Frequent hospitalizations, treatment-related fatigue, and functional limitations commonly restrict daily activities and reduce overall well-being. Physical inactivity is particularly prevalent among dialysis patients and has been identified as an independent predictor of increased mortality and poor clinical outcomes.^1^ Furthermore, complications such as anemia, muscle fatigue, muscle atrophy, and reduced aerobic capacity further limit physical activity, increase the risk of falls, and contribute to diminished QoL. Consequently, physical fitness has emerged as an important determinant of survival and functional status in individuals with ESKD.^2^ QoL among dialysis patients varies significantly across regions due to differences in socioeconomic conditions, healthcare infrastructure, and access to dialysis services.^3^ In the Middle East, individuals undergoing dialysis often experience poorer overall health outcomes compared with global averages.^4^ In Qatar specifically, factors such as sleep disorders and depression have been shown to further reduce QoL and increase the perceived burden of treatment.^5^ Although exercise interventions have been explored as a potential strategy to improve physical and psychosocial outcomes, the evidence remains inconsistent. For example, a randomized controlled trial involving 87 participants found no significant improvement in physical QoL after a 3-week exercise intervention, although important social and environmental domains were not assessed.^6^

Intradialytic resistance exercise (IRE) has been increasingly recognized as a beneficial intervention for hemodialysis patients. Previous studies have demonstrated that medium- to high-intensity intradialytic resistance training (IRE) can safely improve muscle strength, muscle mass, and dialysis efficiency in patients with sarcopenia.^7^ Similarly, a 6-month resistance training program significantly improved muscle strength and physical performance in a multicenter retrospective study of hemodialysis patients.^8^ Despite these benefits, the integration of structured exercise programs within dialysis care remains limited. Another common complication affecting dialysis patients is muscle cramping, which occurs in up to 79% of individuals undergoing hemodialysis and frequently develops during the final hour of treatment.^9^ Preliminary evidence suggests that resistance-based interventions, including stretching exercises, may reduce muscle cramps by lowering electromyography (EMG) activity through stimulation of the Golgi tendon organs.^10^ However, empirical evidence specifically examining the effects of IRE on muscle cramps remains scarce.

In addition to physical symptoms, dialysis adequacy plays a crucial role in determining patients’ QoL. Inadequate dialysis has been associated with poorer physical and psychological outcomes, whereas improved dialysis adequacy may enhance patient well-being.^11^ While intradialytic exercise has been suggested as a potential strategy to improve dialysis efficiency and physical function, its implementation in routine clinical practice remains limited. Nevertheless, international guidelines such as the 2016 Kidney Disease Outcomes Quality Initiative (KDOQI)^12^ and national recommendations in Qatar advocate the incorporation of physical exercise into dialysis care.

Despite recommendations, hospitals often exclude exercise from dialysis treatment plans due to limited staff expertise^13^ and insufficient family support.^14^ Also, IRE during dialysis faces challenges like access to exercise equipment.^15^ While TheraBand’s affordability and portability support home exercise, its feasibility for Qatar’s dialysis patients remains unstudied.

TheraBand IREs were performed targeting the ankle, knee, hip, and non-dialysis arm, and cool-down exercises. TheraBand-based isotonic exercises were selected because they offer a safe and adaptable form of resistance training that can be performed during hemodialysis without interfering with treatment. These exercises provide controlled muscle activation with minimal joint strain, making them appropriate for patients who commonly experience fatigue, weakness, and reduced mobility. Their adjustable resistance allows individualized progression, promoting improvements in strength, functional capacity, and overall well-being.

In Al Khor Hospital in Hamad Medical Corporation, dialysis patients engage less in physical activity, which may be due to a lack of motivation, poor socioeconomic status, may lack support, affecting their ability to adhere to an exercise regimen.^16^ Also, in Al Khor, most patients are laborers with limited economic resources and family support, hindering independent exercise initiation.

TheraBand IREs offer a practical solution for intradialytic exercise in hemodialysis units, tailored to patients’ needs to enhance adherence.^17^ No studies have explored IRE effects on QoL, dialysis adequacy, or muscle cramps in Qatar’s dialysis population. Therefore, this study aims to identify the effect of IRE on dialysis patients residing in Qatar.

## 2. METHODOLOGY

### 2.1 Study design and oversight

This study utilized a quasi-experimental pre–post design to evaluate the QoL in hemodialysis patients, where QoL refers to a broad, multidimensional construct that captures an individual’s physical abilities, psychological well-being, social relationships, environmental well-being, and overall perception of health and life satisfaction, which was effectively and easily used to measure the QoL in hemodialysis patients.^18^

These domains are particularly relevant in the hemodialysis population, who often experience fatigue, functional limitations, and symptom burden. QoL was assessed before and after implementing an intradialytic exercise intervention using isotonic exercises with TheraBands.

**Figure 1.**
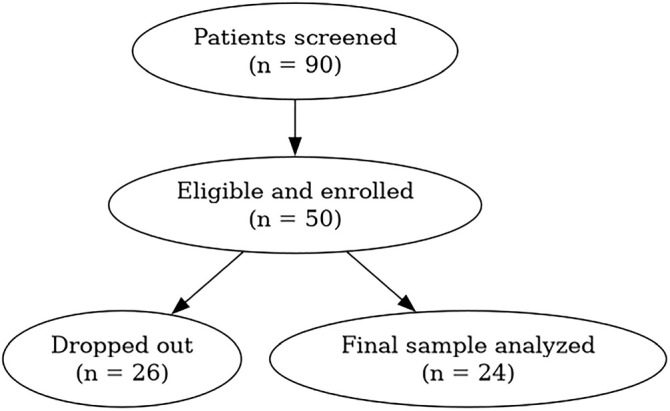
Flowchart of participant recruitment and study completion.

The duration of the study is from July 2023 to December 2023. Baseline data for QoL and physical function were collected in June 2023, immediately before the initiation of the exercise intervention. Preintervention (3-month) baseline data for the secondary outcomes—dialysis adequacy and muscle cramps—were gathered from April to June 2023. The total number of patients enrolled was 50 (Figure 1). Twenty Six patients dropped out due to transfer of treatment to another facility, transfer to peritoneal dialysis treatment, missed continuous 2 weeks of exercise, or death.

Following completion of the 3-month intervention, data for the primary outcome were collected in September 2023. Due to feasibility considerations, secondary outcomes (dialysis adequacy and muscle cramps) were assessed only during the 3-month intervention period. Postintervention data were collected in October–December 2023. Postintervention data for QoL and physical function were subsequently collected in January 2024. Data on QoL were collected at three time points—before the intervention, after the 3-month intervention, and postintervention—to evaluate changes in QoL resulting from the exercise program (Table 1).

The QoL data are collected using the WHO QoL – BREF questionnaire. Each item of the WHO QoL – BREF is rated on a 5-point Likert scale, with higher scores indicating a better perceived QoL. The instrument includes 26 items organized into four domains: Physical Health (7 items), Psychological Health (6 items), Social Relationships (3 items), and Environment (8 items). In addition, the questionnaire includes two general items that assess overall QoL and overall health. Missing data were managed per WHO guidelines, discarding assessments with over 20% missing data and substituting means for missing items within domains when appropriate. Detailed scoring procedures and calculation methods for domain scores and transformations are provided in the official WHO QoL – BREF manual.^19^

### 2.2 Sample size

The sample size calculation was performed based on detecting a mean change in QoL related to the burden of kidney disease from baseline to 3 months. The calculation was based on the expected mean change of 14.6 ± 18.5, as reported by Zhang et al.^6^ The required sample size was estimated using the formula for detecting a mean difference in a paired design: *n* = (Zα/2 + Zβ)^2^ × σ^2^/d^2^, where Zα/2 = 1.96 for a two-sided significance level of 0.05, Zβ = 1.28 corresponding to 90% statistical power, σ = 18.5 represents the standard deviation of the change score, and *d* = 14.6 is the expected mean difference. Based on these assumptions, the minimum required sample size was 17 participants to detect a statistically significant change in the QoL score. To account for an anticipated dropout rate of approximately 40%, the sample size was increased to ~30 participants to ensure adequate power for detecting a statistically significant change in the QoL score.

### 2.3 Study setting/location

The study was conducted at the Al Khor Dialysis Unit under Hamad Medical Corporation, where exercise interventions were provided to patients seated in their dialysis chairs during treatment.

### 2.4 Study population

Eligible participants were more than 18 years old, diagnosed with ESKD, and willing and able to participate in the rehabilitation program.

### 2.5 Inclusion and exclusion criteria

Eligible patients were ≥18 years, on dialysis for ≥3 months, and receiving dialysis at least twice weekly. Exclusions included motor/sensory disorders, frequent dialysis complications, hospitalization causing ≥2 weeks of missed exercise, or incomplete WHO QoL – BREF (>20% missing data).

### 2.6 Intervention

QoL was evaluated using the WHO QoL – BREF questionnaire before, after 3 months, and after a 6-month exercise program, alongside physical function tests (10-m walk, 30-second sit-to-stand; source: electronic health records). The program, conducted during the first 2 hours of dialysis, included baseline functional assessments performed on the first day before the start of dialysis. These assessments consisted of the 10-m walk test, which measures gait speed and functional mobility, and the 30-second sit-to-stand test, which evaluates lower-limb strength and endurance by counting the number of times a patient can rise from a chair within 30 seconds. These measures were used to establish preintervention physical performance levels.

Physiotherapists monitored blood pressure, heart rate, and the Borg RPE (Rating Perceived Exertion) scale, ensuring safety and proper technique. Patients could rest if discomfort occurred, and staff competence was regularly assessed (Supplementary Material).

### 2.7 Ethical approval

This study was reviewed and approved by the Institutional Review Board (IRB) and the Medical Research Center (MRC) of Hamad Medical Corporation (HMC), Doha, Qatar (MRC number: MRC-01-22-464). All procedures involving human participants adhered to the ethical standards of the institutional research committee and were conducted in accordance with the principles outlined in the Declaration of Helsinki. Written informed consent was obtained from all participants before data collection.

### 2.8 Statistical analysis

Discrete variables were presented as frequencies and proportions, while continuous variables were expressed as mean ± standard deviation (SD). Mean scores for QoL and physical function questionnaires were calculated. An intention-to-treat analysis was done for this study. To determine the differences in mean scores over time, the study employed repeated measures analysis of variance (ANOVA). Analysis of covariance (ANCOVA) was utilized to examine the relationship between scores and baseline values, along with potential predictors such as age, gender, education, comorbidities, and duration of hemodialysis. All statistical analyses were conducted using STATA version 17 software.^20^ All tests were two-tailed, with statistical significance established at *P* < 0.05.

## 3. RESULTS

Table 2 illustrates the demographic and socio-economic characteristics of a sample population of 24 individuals. The average age was 59.9 ± 14.9 years. The sample was mainly male (71%) and female (29%). Most participants were married (96%), with 79% unemployed and 21% employed. In terms of living arrangements, 71% lived with their families.

Educationally, 25% had completed 12th grade, 42% had education from primary to higher secondary levels, 21% were graduates, and 13% had no formal education. Income-wise, 58% earned less than or equal to Qatar riyal 5000 per month, while 42% earned more than 5000 per month.

**Figure 2.**
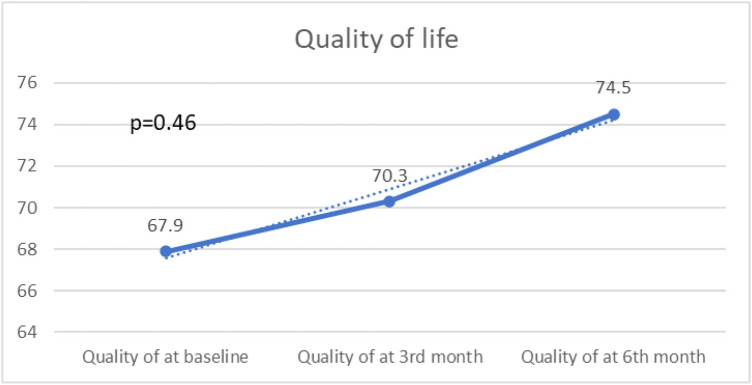
Changes in quality-of-life scores over time.

Figure 2 presents the average value of QoL scores at different time points: baseline, after 3 months, and after 6 months. At baseline, the average QoL score was 67.9 ± 20.9. After 3 months, the average score increased to 70.3 ± 17.2 and in 6 months, the average score further increased to 74.5 ± 15.8; however, the change was not statistically significant.

Table 3 displays the relationship between QoL scores at baseline, after 3 months, and after 6 months, and factors such as gender, education, living situation, and income. Regarding gender, females tended to report slightly better QoL than males at all time points, but the gap was not large enough to be considered important. Education levels did not show any meaningful or statistically significant relationship with QoL at baseline, 3 months, or 6 months. Although the scores varied across the different education groups, these differences were not big or consistent enough to indicate that education had a real impact on QoL in this sample. For living situations, people living with family generally showed better QoL than those living alone, but these differences were not statistically significant. In terms of income, individuals with higher income tended to report better QoL than those earning less, but again, the differences were not strong enough to be meaningful.

Table 4 illustrates the association between sociodemographic factors and QoL using generalized estimation equations, providing both unadjusted and adjusted coefficients, 95% confidence intervals (CI), and P-values. For QoL after 3 months, the unadjusted coefficient is 2.45 (95% CI: −5.93, 10.83; *P* = 0.566), and after 6 months, it is 6.62 (95% CI: −1.76, 15; *P* = 0.121), indicating no significant change from baseline. None of the sociodemographic variables, including age, gender, education, employment, living with family, and income, showed any statistically significant associations with QoL in either the unadjusted or adjusted models.

**Figure 3.**
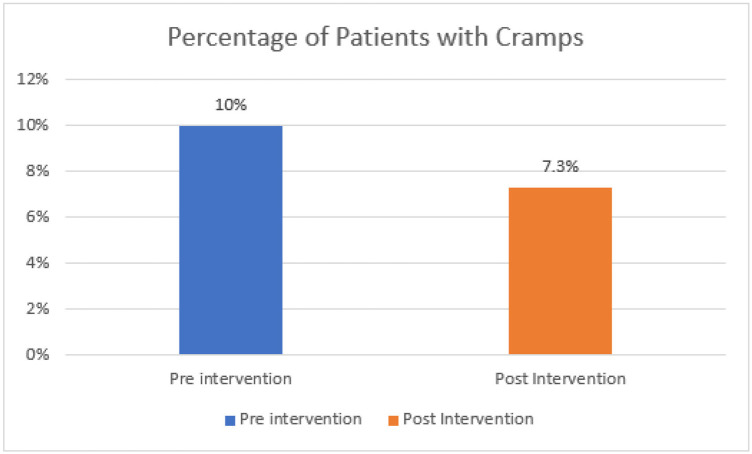
Percentage of patients experiencing cramps before and after the intervention.

For the secondary outcome related to cramps, the data in Figure 3 shows the percentage of patients with cramps at baseline and post intervention. Initially, 10% of patients reported cramps at baseline. Following the intervention, the percentage reduced to 7.3%, indicating a reduction in the episodes over time with exercise.

Before the intervention, the mean Kt/V was analyzed. Kt/V is a standardized measure of dialysis adequacy that reflects how effectively urea is cleared during a hemodialysis session. It is calculated from three components: K (dialyzer clearance of urea), t (duration of dialysis), and V (the patient’s urea distribution volume, approximately equal to total body water). Higher Kt/V values indicate better removal of metabolic waste and more efficient dialysis. In this study, the mean Kt/V values demonstrated a gradual improvement before the intervention, increasing from 1.7 ± 0.30 in the first month (April) to 1.7 ± 0.33 in the second month (May), before showing a slight decline to 1.6 ± 0.21 in the third month (June). These fluctuations may reflect routine monthly variations in dialysis efficiency influenced by factors such as blood flow rate, dialyzer performance, patient hydration status, and treatment adherence. Although the changes were modest, the overall values remained within the clinically acceptable range for adequate dialysis.

**Figure 4.**
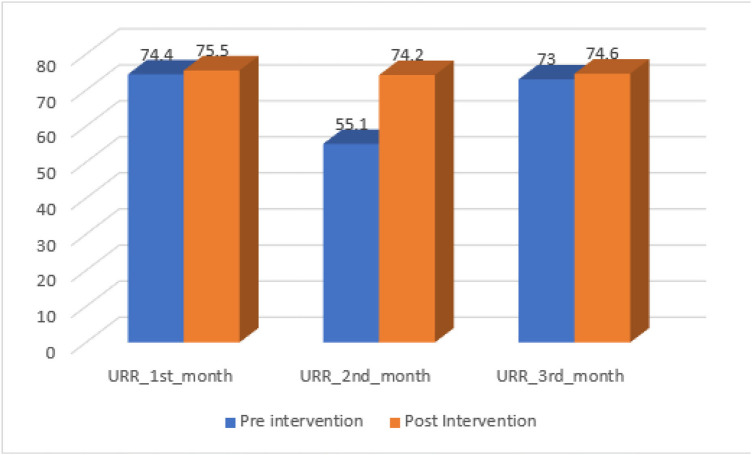
Mean Urea Reduction Ratio (URR) across three consecutive months before and after the intervention. Blue bars indicate pre-intervention monthly values, while orange bars represent values after three months of intervention. The figure illustrates trends within each phase rather than direct comparisons between corresponding months.

Following the intervention, the mean Kt/V reached 1.67± 0.22 in the first measurement (October) and remained almost stable at 1.68 ± 0.24 in the second (November) and 1.65 ± 0.26 in the third measurement (December; see Figure 4).

Postintervention, the median Kt/V was 1.7 (IQR: 1.7, 1.7) in the first measurement, 1.6 (IQR: 1.5, 1.6) after 3 months, and 1.9 (IQR: 1.8, 1.9) after 6 months.

Concurrently, the mean urea reduction ratio (URR) was also measured initially. The URR is another commonly used indicator of dialysis adequacy and represents the percentage reduction in blood urea concentration during a single dialysis session. Higher URR values indicate more efficient removal of urea and overall better dialysis performance. In this study, the mean URR values demonstrated notable fluctuations. Before the intervention, URR initially averaged 74.4 ± 5.3% (April), then dropped markedly to 55.1 ± 33.6% in the second month (May) before improving again to 73.0 ± 6.6% by the third month (June). Such variability may reflect temporary changes in clinical or technical factors, including differences in blood flow rate, vascular access performance, patient fluid status, or session-to-session adherence. Postintervention, the URR values were more stable, beginning at 75.5 ± 4.3% (October), slightly decreasing to 74.2 ± 6.0% (November), and leveling off at 74.6 ± 5.6% by the third assessment (December; Figure 3).

**Figure 5.**
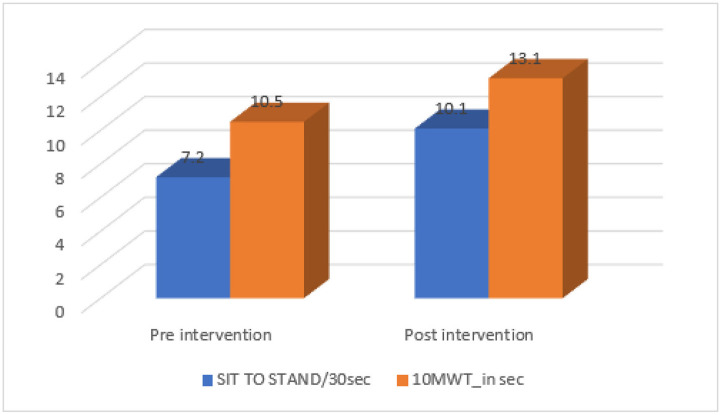
Physical performance before and after intervention.

The study evaluated physical performance using the sit-to-stand test and 10-m walk test. Preintervention, participants averaged 7.2 ± 2.9 sit-to-stand repetitions, improving to 10.1 ± 4.5 postintervention, reflecting enhanced lower body strength and endurance. Conversely, 10-m walk test times slightly increased from 10.5 ± 12.9 to 13.1 ± 24.1 seconds, suggesting no significant improvement in walking speed (Figure 5).

## 4. DISCUSSION

The study investigated IRE’s effect on QoL in Qatar’s hemodialysis patients, finding QoL improvements after 6 months. Although non-significant, it is consistent with research indicating gradual exercise benefits.^21^ Regular intradialytic exercise is vital for dialysis care, suggesting long-term benefits beyond short-term observation.

In this study, women had slightly higher QoL scores than men after both 3 and 6 months (78.6 ± 9.4 vs. 66.9 ± 18.7 at 3 months), although the difference was not statistically significant. Despite this, the improvement in women’s QoL scores could be meaningful, as women with kidney failure typically experience lower QoL compared to men. This suggests that exercise could positively impact the QoL for female hemodialysis patients.^22^ This suggests that structured exercise programs may help mitigate gender-based disparities in QoL, although further studies with larger sample sizes are needed to confirm this.

The results of this study also supported the findings of previous research on the relationship between exercise and dialysis adequacy. Lamia et al.^11^ reported that improving dialysis adequacy can enhance multiple QoL components. Our study found that IRE, while not significantly improving QoL scores in the short term, may have a positive impact on dialysis adequacy, which in turn could contribute to improved QoL over time.

The study also provides valuable insights into the feasibility of IRE for hemodialysis patients in Qatar. The use of TheraBand exercises, which are relatively low-cost and portable, can make it easier for patients to incorporate exercise into their daily routines.^23^ However, the study also highlights the importance of addressing barriers such as lack of motivation and limited family support, which can hinder adherence to an exercise regimen.

The reason for the high dropout rate was largely attributable to foreign workers who went on vacation, which fell during this 6-month duration, and hospital transfer, sickness, death, and sustainability to a lesser extent.

The percentage of patients who experienced cramps was 13% after 3 months of the exercise regimen. Postintervention, a decrease to 7.3% was observed, indicating a positive impact of the intervention on cramping. Participants reported reduced cramps and increased motivation, which led to a desire to continue exercising independently. However, they valued the physiotherapist’s support and guidance. This study aligns with another quasi-experimental study, which found that there is a reduction of cramping with IRE.^24^

Dialysis efficacy was calculated using the equation of single-pool Kt/V. All blood samples for examination of serum concentrations were drawn predialysis. Kt/V, the fractional urea clearance, is the most precise and tested measure of the dialyzer effect on patient survival and is the most frequently applied measure of the delivered dialysis dose.^25^ According to the Renal Physicians Association (RPA) Instruction in 1993, at least URR > 65% and Kt/V > 1.2 are considered for dialysis adequacy. The National Kidney Foundation (NKF)-Dialysis Outcomes Quality Initiative (DOQI) also identified these criteria in 1997 and changed the Kt/V value to 1.4 in 2006, and identified Kt/V > 1.2 as the minimum acceptable. In accordance with the previous literature, this study also proved that there was an improvement in dialysis adequacy with intradialytic exercise.

Zelko et al. found that IRT improved both hip abductors (HA) and hip flexor (HF) strength in hemodialysis patients.^26^

Given the high risk of falls, movement disabilities, and immobilization in this population, maintaining muscle function is crucial. We observed improvements in muscle strength, positive impact of the intervention on physical performance and mobility as evidenced by improvements in the sit-to-stand and 10 m walk test. All patients completed 50-56 exercise sessions. There were no adverse events reported during the study. Additionally, participants reported significant improvements in their ability to perform daily activities, such as hand grip, walking, and managing knee pain. Participants exhibited increased lower-body strength and endurance, suggesting that the intervention was effective in enhancing functional capacity. To improve the effectiveness of interventions and reach the full potential of patients’ adaptability, it is necessary to create personalized activity prescriptions for every patient.

### 4.1 Limitations

One limitation of this study is the relatively small sample size. A larger sample size would have allowed for a more robust analysis of the relationship between IRE and QoL, dialysis adequacy, and muscle cramps. Second, the study did not include a control group, making it difficult to isolate the specific effects of IRE. Third, although this study focused primarily on physical and QoL outcomes, it did not include a direct assessment of patients’ psychological state or exercise-related psychological constructs such as self-efficacy. This represents an important limitation, as psychological factors such as anxiety, depression, and perceived control can also influence how patients participate in intradialytic exercise programs and how they perceive improvements in QoL. Additionally, while the selected TheraBand isotonic exercises targeted strength and functional mobility, other important domains, such as balance ability and body composition, were not evaluated. Measurements such as postural stability, muscle mass, and fat-free mass could provide a more comprehensive picture of functional changes resulting from intradialytic exercise.

Future studies should address these limitations to provide further evidence on the benefits of IRE for hemodialysis patients with a larger sample size. Lastly, the study only assessed QoL for 6 months, and longer-term follow-up would be needed to evaluate the sustainability of the effects of IRE.

## 5. CONCLUSION

The study demonstrated that exercise intervention using isotonic exercises with TheraBands during dialysis sessions led to modest improvements in the QoL and physical performance of hemodialysis patients. However, these changes were not statistically significant. While the findings suggest that structured exercise programs can have potential benefits in enhancing patient outcomes, the lack of significant results indicates the need for larger, long-term studies to better understand the full impact of exercise on QoL, physical function, and overall health in this population. Tailoring interventions to individual patient needs may also enhance effectiveness.

## DATA AVAILABILITY

The data supporting the findings of this study are available from the corresponding author upon reasonable request.

## ETHICAL APPROVAL

This study was reviewed and approved by the Institutional Review Board (IRB) and the Medical Research Center (MRC) of Hamad Medical Corporation (HMC), Doha, Qatar. MRC Number-MRC-01-22-464. All procedures involving human participants adhered to the ethical standards of the institutional research committee and were conducted in accordance with the principles outlined in the Declaration of Helsinki. Written informed consent was obtained from all participants prior to data collection.

## AUTHOR CONTRIBUTIONS

All authors contributed to the study conception, design, data collection, analysis, and manuscript preparation. All authors reviewed and approved the final manuscript.

## FUNDING

This research did not receive any specific grant from funding agencies in the public, commercial, or not-for-profit sectors.

## ACKNOWLEDGEMENT

The authors would like to express their sincere gratitude to all the participants who took part in this study for their time and cooperation. We also acknowledge the support of the dialysis unit staff for their cooperation. We extend our appreciation to our colleagues and institutional leadership for their guidance and support throughout the study.

## DISCLOSURE OF AI USE

The authors acknowledge the use of artificial intelligence (AI) tools for assistance with grammar correction and language enhancement. No AI tools were used in the design, data collection, analysis, or interpretation of the study. The authors confirm full responsibility for the accuracy and integrity of the work.

## CONFLICT OF INTERESTS

The author(s) declare that there is no conflict of interest.

## SUPPLEMENTARY MATERIAL

**Improved Joint and Muscle Range of Motion in Preparation For Resistance Exercises**



## Figures and Tables

**Table 1.** Timing of baseline, 3-month, and postintervention data collection for quality of life, muscle cramps, physical function, and dialysis adequacy across the study period.

Period of data collection	Quality of life	Muscle cramps	Physical function	Dialysis adequacy
Baseline data	June 2023	3-month data (April–June 2023)	June 2023	3-month data (April–June 2023)
After 3 months	September 2023	-	-	-
Postintervention data	January 2024	Data from October–December 2023	January 2024	Data from October–December 2023

**Table 2.** Participant characteristics.

Variables	Level	Value
*N*		24
Age, mean (SD)		59.9 (14.9)
Gender	Female	7 (29%)
	Male	17 (71%)
Married	No	1 (4%)
	Yes	23 (96%)
Employment	No	19 (79%)
	Yes	5 (21%)
Living with family	No	7 (29%)
	Yes	17 (71%)
Education	12^th^	6 (25%)
	Primary to higher secondary	10 (42%)
	Graduate	5 (21%)
	None	3 (13%)
Income (income/month)	≤5000	14 (58%)
	>5000	10 (42%)

**Table 3.** Association with socio-demographic variables at different time points.

Variables	*N*	Quality of life at baseline, mean (SD)	Quality of life after 3 months, mean (SD)	Quality of life after 6 months, mean (SD)
**Gender**				
Female	7	72.9 (24.3)	78.6 (9.4)	76.8 (13.4)
Male	17	66.2 (20.1)	66.9 (18.7)	73.5 (17.0)
*P* value		0.51	0.13	0.66
**Education**				
12^th^	6	66.7 (21.9)	75.0 (11.2)	79.2 (17.1)
Primary to higher secondary	10	62.5 (25.7)	67.5 (19.7)	71.3 (16.7)
Graduate	5	71.9 (6.3)	62.5 (19.8)	70.0 (11.2)
None	3	83.3 (7.2)	83.3 (7.2)	83.3 (19.1)
*P* value		0.51	0.34	0.54
**Living with family**				
No	7	75.0 (21.7)	62.5 (17.7)	67.9 (17.5)
Yes	17	64.8 (20.5)	73.5 (16.5)	77.2 (14.8)
*P* value		0.29	0.16	0.20
**Income**				
≤5000	14	65.2 (22.0)	65.2 (19.7)	70.5 (16.7)
>5000	10	72.2 (19.5)	77.5 (9.9)	80.0 (13.4)
*P* value		0.44	0.084	0.15

**Table 4.** Association with sociodemographic factors with quality of life using generalized estimation equations.

Variables	Unadjusted		Adjusted	
	Coeff. 95% (CI)	*P* value	Coeff. 95% (CI)	*P* value
**Quality of life**				
Baseline	Ref		Ref	
After 3 months	2.45 (−5.93, 10.83)	0.566	2.32 (−8.05, 12.69)	0.656
After 6 months	6.62 (−1.76, 15)	0.121	6.49 (−3.88, 16.86)	0.216
Age	0.24 (−0.11, 0.58)	0.182	0.04 (−0.41, 0.49)	0.862
**Gender**				
Female	Ref		Ref	
Male	−7.22 (−18.42, 3.98)	0.206	−4.04 (−20.46, 12.38)	0.625
**Employment**				
No	Ref		Ref	
Yes	3.03 (−9.78, 15.85)	0.64	2.35 (−13.63, 18.32)	0.77
**Living with family**				
No	Ref		Ref	
Yes	3.51 (−7.9, 14.94)	0.546	−2.4 (−14.35, 9.54)	0.689
**Education**				
12^th^	Ref		Ref	
Primary to higher secondary	−6.53 (−18.53, 5.48)	0.287	−4.17 (−18, 9.67)	0.549
Graduate	−5.73 (−19.98, 8.52)	0.43	−9.06 (−23.32, 5.2)	0.209
None	9.72 (−6.72, 26.16)	0.246	3.97 (−17.61, 25.55)	0.714
**Income**				
≤5000	Ref		Ref	
>5000	9.65 (−0.23, 19.54)	0.056	8.47 (−4.98, 21.91)	0.213
