# Supplementary material for: Effect of intradialytic resistance exercise on quality of life of hemodialysis patients in Hamad Medical Corporation: A quasi-experimental study
Source: Qatar Med J. 2026 Jun 15;2026(2):33. doi: 10.5339/qmj.2026.33 (PMC13436420; doi:10.5339/qmj.2026.33)
Supplement: Supplementary file 1 [file qmj-2026-02-033.supp1.docx]

**Improved Joint and Muscle Range of Motion in Preparation For Resistance Exercises**

| **Exercise Title** |  | **Instructions** |
| --- | --- | --- |
| Ankle Pumps |  | Point your toes down by moving the ankle then raise them up back to starting position. |
| Eversion/Inversion |  | Move your ankle from side to side then back to starting position. |
| Knee flexion/Extension |  | Keep the heel of your leg on the bed. Then slide the heel toward your buttocks as far as you comfortably can, extend your leg back to starting position. |
| Hip flexion/extension |   . | Lie in bed. Bend one leg. Keep your other leg straight on the bed. Tighten your thigh muscle and lift your straight leg as high as you can. Slowly lower the leg back to the bed. |

| Hip Abduction/adduction |  | Start with your feet slightly apart. Keeping your knee and foot pointing toward the ceiling, slowly slide one leg out to the side. Slide your leg back to its starting position. |
| --- | --- | --- |
| Shoulder flexion/extension |  | Raise your arm up while keeping the elbows straight. Then bring it down to the starting position. |
| Shoulder abduction/adduction |  | Move your arm away from your body while maintaining the elbow in straight position. Bring back your arm to the side of your body. |
| Elbow flexion/extension |  | Flex your arm straight out in front of you, keeping your wrist straight. Slowly bend your arm toward you as far as you can; then straighten it until it is back to its original position. |
| Wrist flexion/extension |  | Bend your wrist forward and backward. |
| **THERABAND EXERCISES** | | |
| Aims to strengthen muscles and improve flexibility. Increase muscle endurance and tone. |   Plantar flexion/ eversion/inversion | Place one foot in the middle of the resistance band and hold both ends of it with your hands.  Extend your leg and point your toes towards the ceiling, then point your toes forwards towards the ground.  Return to the starting position  In same position, point your toes out towards the side stretching the band and point them back in. |
| Knee extension |  | Place one foot in the middle of the resistance band and hold both ends of it with your hands. Bend your knee towards you, then straighten it back out in front of you before returning to your starting position. |
| clamshell |  | Tie the band around your thighs, while keeping the knees bent and feet together, stretch the band by opening the legs. Return to starting position. |
| Abduction |  | Tie the band around your thighs. Move one leg to the side while stretching the band, return to starting position and do same for the other leg. |
| Hip flexion |  | Place one foot in the middle of the resistance band and hold both ends of it with your hands. Raise your leg up while keeping the  knee in straight position. Return to starting position. |
| Stretching exercises aim to Improve your performance in physical activities  Decrease your risk of injuries  Help your joints move through their full range of motion  Increase muscle blood flow  Enable your muscles to work most effectively  Improve your ability to do daily activities | Hamstrings stretch  | Keep your knee straight and toes pointing to ceiling, move your body forward towards your feet until you feel the stretch at the back of your knee, hold for 10 seconds then relax. |
| Quadriceps stretch |  | Flex your knee as far back as you can until you feel the stretch inform of your thigh, hold for 10 seconds then relax. |
| Gastrocnemius stretch |  | While keeping the knee straight, move your toes up to wards your shin, hold for 10 seconds then relax. |
